# Capturing Family Planning Community Service Data into National Health Management Information Systems: A Five-Country Review of Tools for Community Health Workers and Pharmacies and Drug Shops

**DOI:** 10.12688/gatesopenres.16397.1

**Published:** 2026-09-01

**Authors:** Aurélie Brunie, Lucy Wilson, Elena Lebetkin, Lawrence Anyanwu, Mathieu Bougma, Valério Govo, Binod Joshi, Rogers Kagimu, Fredrick Makumbi, Sarah Nabukeera, Funmilola M. OlaOlorun, Aluka Terpase, Trinity Zan

**Affiliations:** 1 FHI 360 Washington, Washington, District of Columbia, USA; 2 Rising Outcomes, Hillsborough, NC, 27516, USA; 3 FHI 360, Durham, North Carolina, USA; 4 Federal Ministry of Health, Reproductive Health Division, Nigeria, Abuja, Nigeria; 5 Ministry of Health and Public Hygiene, Family Planning Department, Burkina Faso., Ouagadougou, Burkina Faso; 6 Consultant to FHI 360, Mozambique, Maputo, Mozambique; 7 Avenir Health, Nepal, Kathmandu, Nepal; 8 Ministry of Health, Division of Health Information Management and Division of Reproductive and Infant Health, Uganda, Kampala, Uganda; 9 School of Public Health, Makerere University, Uganda, Kampala, Uganda; 10 College of Medicine, University of Ibadan, Nigeria, Ibadan, Nigeria; 11 Evidence for Sustainable Development Systems in Africa (EVIHDAF), Yaoundé, Cameroon; 12 Health Information Systems Nigeria, Abjua, Nigeria

**Keywords:** health information systems; community health workers; pharmacies; drug shops; Burkina Faso; Mozambique; Nepal; Nigeria; Uganda

## Abstract

**Background:**

Inclusion of data on services provided by community health workers (CHWs) and pharmacies and drug shops (PDS) in national health management information systems (HMIS) is important for planning and decision-making related to these family planning high impact practices (HIPs) and to family planning programming more broadly. Yet little is known on the extent to which information on CHW and PDS family planning activities effectively feeds into HMIS.

**Methods:**

We conducted a review of HMIS tools in Burkina Faso, Mozambique, Nepal, Nigeria and Uganda to examine availability of family planning data elements on CHWs and PDS. Participatory discussions held as part of a subsequent webinar generated considerations for advancing measurement of these two HIPs through national HMIS.

**Results:**

CHW registers and reporting forms include data elements on method provision and referrals in four of the five countries, but only include data elements on counseling in one country. Data flows vary substantially across countries. There is very limited integration of PDS data into the HMIS. Participatory discussions coalesced around a recommendation to capture information on CHW method provision and for leaving additional indicators on counseling and referrals at the discretion of countries. For PDS, discussions centered on challenges and experiences with enabling private sector reporting.

**Conclusion:**

The review and discussions highlight the value and complexities of integrating data on community sources into the HMIS and the need for context-specific solutions. Interest and opportunities exist to align CHW indicators across settings. For PDS, more discussion on how to incentivize reporting and on feasible reporting platforms and processes is needed.

**List of abbreviations**
ASBC: Agents de Santé à Base Communautaire
APS: Agentes Polivalentes de Saùde
CHW: Community Health Worker
FCHV: Female Community Health Volunteers
HIP: High Impact Practice
HMIS: Health Management Information System
PDS: Pharmacies and Drug Shops
VHT: Village Health Teams

## Introduction

In low- and middle-income countries faced with high unmet need for family planning and health-worker shortages, community health workers (CHWs) and pharmacies and drug shops (PDS) are important sources of family planning information and contraceptive methods, in part due to their easy access and convenience. ^1^^–^^3^ CHWs and PDS are recognized as proven and promising family planning high impact practices (HIPs), respectively. ^4^^,^^5^ HIPs are evidence-based practices vetted by experts and identified based on demonstrated impact on contraceptive use, as well as scalability, sustainability, cost-effectiveness and applicability in a wide range of settings. ^6^ Since the beginning of the HIP partnership, a global collaborative started in 2010, the promotion and implementation of HIPs has grown steadily in low- and middle-income countries.

National health management information systems (HMIS) are essential for planning, management, and decision-making at national and subnational levels. Uses of the HMIS include measuring progress against goals, monitoring products and services, and informing resource allocation. At the HMIS level, data from CHWs and PDS are important for monitoring HIP implementation and scale-up, as well as to provide a comprehensive picture of the family planning service landscape in a country. ^7^^,^^8^

Mechanisms for collecting, reporting and analyzing data on family planning services provided at public sector health facilities tend to be well-established although there is variation in data flows and reporting formats. Routine data are typically captured in pre-printed registers at health facilities, then aggregated from registers on a monthly basis and transmitted in summary forms to a higher level. Aggregated totals are generally entered electronically using software like DHIS2 at some point in the reporting chain and available at higher levels, including the national level.

Despite the well-established data processes within public-sector facilities, there are challenges to integrating data from CHW and PDS data into the national HMIS. PDS and some CHWs are part of the private sector, which is not consistently captured in HMIS across low- and middle-income countries. ^7^ The number of PDS and CHWs is likely to greatly exceed that of health facilities, and as they are more diffuse than public sector facility-based providers, training and equipping them to collect and report HMIS data requires a substantial investment. ^9^ Models for community-based programs vary, with differences in skills, responsibilities, levels of training and supervisory structure across, and sometimes within, countries. ^10^^,^^11^ Additionally, non-governmental organizations and donors have historically played a large role in supporting implementation of community-based services through CHWs and PDS. ^1^^,^^12^ This has resulted in siloed and diverse reporting systems, including differences in the types and sources of data collected, tools, data flows, and the extent to which data are assimilated into the national HMIS operated by the Ministry of Health. ^7^^,^^9^^,^^12^ Furthermore, modifications to national HMIS tools and indicators are typically made through established Ministry of Health processes rather than on an ad hoc basis. As a result, incorporating new data elements often depends on periodic HMIS review cycles, during which proposed changes are reviewed and approved through technical and stakeholder consultation processes.

Analysis of whether and how data elements from CHWs and PDS are captured into the national HMIS is important to understand the extent to which countries can effectively steward implementation and scale-up of these two HIPs, as well as access comprehensive information on family planning service utilization in their settings. We undertook a review of data collection and reporting tools across five countries to examine availability of family planning data elements on CHWs and PDS. Results from this review were presented as part of a global webinar aimed at advancing measurement of the coverage and service utilization of HIPs through national HMIS. ^13^ The webinar included break-out groups as a step towards informing potential recommendations for global indicators for CHWs and PDS. This manuscript summarizes findings from the five-country review of reporting tools and outlines key considerations for routine measurement of CHW and PDS family planning activities that emerged from the webinar.

## Methods

The five countries were selected because they were part of a larger study to develop and test measures for implementation and scale-up of four service delivery HIPs, which included CHWs and PDS. The review process was informed by another recent review of country tools related to two facility-based HIPs, immediate postpartum family planning (IPPFP) and postabortion family planning (PAFP). ^14^ One key difference was that the earlier review was framed around existing global recommendations for IPPFP/PAFP indicators, ^15^ whereas there are no agreed-upon recommendations for CHWs/PDS. Another difference is that IPPFP and PAFP services are provided at a facility, whereas the focus of this review is on services provided in communities. There are differences in data flows across contexts and between CHWs and PDS. That said, as with facility-based routine health information systems, a generic data flow starts with recording of interactions with clients in a register and involves entry of aggregate monthly totals into reporting forms to send to a higher level in the reporting chain. ^9^^,^^16^ Part of this review involved understanding whether and how this information may then enter the national HMIS – at the facility level, or higher. The review proceeded in three steps, described below.

*Obtaining registers and forms:* Starting in November 2023, we gathered CHW and PDS registers, CHW and PDS monthly summary forms and any other summary forms showing data elements related to CHWs and PDS for each country from Ministries of Health through country partners in the larger study. The intent was to identify tools provided or endorsed by the government, as opposed to tools that were developed and used by implementing partners. We aimed to conduct a cross-sectional analysis and requested the most recent version of these documents only. Where necessary, we obtained formal permission from the Ministry of Health to conduct the review. We obtained copies of data collection and reporting tools in formats including Microsoft Excel, Word, PDF, and images. We translated tools in French and Nepali to English using DeepL and Google Translate prior to analysis.

*Summary of CHW/PDS data elements:* We conducted an initial review of the tools to produce comprehensive lists of CHW and PDS data elements - the individual fields or variables captured by each tool (e.g., client age, contraceptive method provided, referral status). We did not review or analyze the service data recorded within those fields. We then compared the lists of data elements across tools to identify the most common thematic categories. Thematic categories refer to groups of data elements that may be formulated differently but capture the same concept. For CHWs, we identified three common thematic categories: family planning counseling, contraceptive method provision, and referrals. For PDS, there were not enough tools to identify common categories of data elements, so we used the same categories as for CHWs. One author (LW) then conducted a second review of all registers and summary forms and, using a data extraction worksheet, mapped available data elements from each country and from each tool to the three categories– specifically paying attention to the distinction between data collection through registers and reporting through summary forms, as well as available disaggregation by age and by method. For Burkina Faso and Mozambique, where we were not able to review registers, if something was recorded in the monthly summary forms, we assumed the information was available in the primary tool (register) since the register provides the basis for reporting forms.

*Cross-checking and validation of results:* We conducted data quality checks through an independent review of registers and summary forms by two authors (EL and AB) to verify the accuracy of the mapping. Additionally, we held calls with other authors representing MOH personnel or other individuals deeply knowledgeable with the HMIS in each country to review and validate country-specific results, using email or phone conversations to clarify data flows as needed.

*Webinar:* We organized a global webinar on 14-15 May 2024 to present the findings and solicit stakeholder perspectives on opportunities and challenges related to harmonizing CHW and PDS indicators. Facilitated breakout group discussions served to explore implications of the results from the review and identify considerations for future measurement efforts within and beyond the countries included in the review. We documented and synthesized key discussion points to inform interpretation of the review findings.

## Results

### Community health workers

For CHWs, we obtained three registers and five summary forms ( Table 1). We were not able to obtain copies of registers for Burkina Faso and Mozambique.

**Table 1.** Registers and summary forms included in the CHW review.

Country	CHW cadre	Register	Summary form (level)	Other sources of info
Burkina Faso	Agents de Santé à Base Communautaire (ASBC)	Not available	Monthly Activity Report: Community (sub-district)	N/A
Mozambique	Agentes Polivalentes de Saùde (APS)	Not available	FP indicator list from UpScale	N/A
Nepal	Female Community Health Volunteers (FCHV)	HMIS 4.2 FCHV Service Register	HMIS 9.1 FCHV Report (supervising facility)	FCHV data in HMIS 9.2–9.5 Facility Reports
Nigeria	Tools apply to all community-based actors	Community-based daily FP register, v 2022	Community HMIS, Community Monthly Summary Form, v 2022	N/A
Uganda	Village Health Teams (VHT)	VHT Household Register	HMIS 105: Health Unit Outpatient Monthly Report (supervising facility)	Meeting with Ministry of Health staff

In Burkina Faso and Nepal, CHWs – called Agents de Santé à Base Communautaire and Female Community Health Volunteers, respectively - transmit reports (their registers or summary forms based on their registers) to their supervising health facilities. The health facility then aggregates data across all the CHWs linked to the facility and enters the totals in a monthly summary form. Data for CHWs are recorded separately from data on services provided at the health facility. In Uganda, CHWs, called Village Health Teams (VHTs), use program-specific or self-designed tools to report their family planning service provision data to their supporting health facility. Data elements on family planning services provided by VHTs are then aggregated with the data from the health facility in the monthly summary form for the health facility, so that in this case, it is not possible to isolate data for VHTs from those for the health facility.

The CHW data tools and flows for Mozambique and Nigeria are based on pilot programs that are currently not rolled out nationally, but that the Ministries of Health confirmed were being integrated into the national HMIS. In Mozambique, CHWs, called Agentes Polivalentes de Saùde, use UpScale, a digital health system strengthening platform, to enter data into an app that feeds directly into DHIS2. ^17^ In Nigeria, the Federal Ministry of Health and Social Welfare has been piloting a Community Health Management Information System (CMHIS) for community-based services, including those of CHWs. The modalities are not fully streamlined, but involve reporting through ward focal points, either with each CHW transmitting a monthly summary form to the local government health office through the ward focal point or with the ward focal point summarizing all community activities into a single form. Data are then uploaded to an electronic CHMIS platform that can be accessed via a dashboard. The CHMIS operates independently from the facility-based DHIS2, but the data elements are aligned and there are plans to integrate the two systems.

The community register and monthly summary form in Nigeria are the only CHW tools from the five countries which include data elements on counseling ( Table 2). CHW registers and monthly summary forms in four countries include data elements on method provision and on referrals. For method provision, data elements in all four countries include both quantities of contraceptive products distributed and numbers of clients. Data elements included in monthly summary forms for referrals vary; in Burkina Faso and Nepal only the number of family planning clients referred is captured, while in Mozambique and Nigeria the forms also capture the reason (i.e., method) for referral.

**Table 2.** Measurement of family planning counseling, contraceptive method provision, and referrals in CHW registers and summary forms.

Country	Family planning counseling		Contraceptive method provision		Referrals	
	Register	Summary form	Register	Summary form	Register	Summary form
Burkina Faso	Unknown	-	X ^A,M^	X ^A,M^	X	X
Mozambique	Unknown	-	X ^A,M^	X ^M^	X	X
Nepal	-	-	X ^M^	X ^M^	X	X
Nigeria	X	X	X ^A,M^	X ^A,M^	X	X
Uganda	-	-	-	-	-	-

‘X’ indicates availability of a data element.

For contraceptive method provision, ^A^indicates availability of disaggregation by age and ^M^indicates availability of disaggregation by method.

The review looked at disaggregation by age and by method for data elements related to method provision. Age is recorded in CHW registers in three countries (Burkina Faso, Mozambique, and Nigeria). Only the monthly summary form in Burkina Faso includes disaggregation of the number of clients receiving each method by age category (19 years old or younger, 20–24 years old, 25 years old and older). In Nigeria, the community summary form includes a count of women and girls using modern contraception in the community broken down into five age groups (10–14, 15–19, 20–24, 25–49, and 50 or older). In Mozambique, the age of each individual client is captured, but data on the number of clients receiving contraceptive methods by age does not appear to be available. All data elements on method provision identified in registers and summary forms are disaggregated by the methods CHWs are authorized to provide in each setting, for all four countries where method provision is captured.

### Pharmacies and drug shops

For PDS, we only obtained one register and one summary form, which were for the Nigeria CHMIS and identical to those reviewed for CHWs.

No data for PDS is collected by the MOH in Burkina Faso and Mozambique. In Nepal, PDS are not recognized as reporting units by the Ministry of Health and Population, though some may report data to a local facility or municipal office. When this happens, data from pharmacies and drug shops are combined with those from health facilities as they get reported and it is not possible to distinguish between the two. Similar to the process for VHTs, data from some drug shop operators in Uganda are included in the monthly summary forms for nearby health facilities. Quantities of commodities distributed are recorded separately for PDS and the health facility in the summary form, but the number of clients receiving contraceptive methods are combined.

## Discussion

Compared to population-based surveys, the HMIS provides more granular and frequent data points and is country-owned. ^18^ Efforts have contributed to strengthening the HMIS for infectious diseases, but some gaps have been documented that affect data quality and use for family planning. ^19^ Yet since the inception of the FP2020 (now FP2030) partnership, there have been substantial improvements in the infrastructure and capacity for generating and reporting family planning data, leading to increased use of routine data by countries to monitor access and availability of family planning services. ^20^ In line with this, effectively integrating data on family planning activities at the community level into the national HMIS can provide valuable information in countries where CHWs and PDS provide a significant proportion of family planning commodities and services. For CHWs, our review of country data collection and reporting tools across five countries underscores the commitment and efforts to build linkages that can bring together facility-based and community-based data. Information on method provision is robust, with disaggregation by method being more frequent than disaggregation by age. Tools also often include information on referrals, but documentation of counseling is less common. Findings on data elements and data flows highlight substantial heterogeneity in the mechanisms used across contexts and point at additional complexities in assimilating CHW data in the national HMIS. CHWs in Burkina Faso, Nepal, and Uganda transmit reports to health facilities, Mozambique is piloting a digital system that feeds CHW data directly into DHIS2, and Nigeria is piloting a parallel CHMIS that has not yet been integrated with DHIS2. Additionally, CHW data are routinely aggregated with health facility data in Uganda, which provides program managers at higher levels with a holistic view of family planning services and activities but limits their ability to examine CHW-specific data to monitor and inform decisions related to CHW programs.

For PDS, we found that there is often no systematic process in place for collecting information from these outlets and that integration of data into the HMIS is therefore very limited to date in the five countries.

These data systems and practices are at different levels of maturity across the countries reviewed: some at pilot, some iterating, others at scale. For our purposes, pilot systems were relevant to understand how ministries have envisioned the best possible data tools and flows for capturing CHW and PDS information, while also being helpful for ministries to understand the actual feasibility and utility of designed systems.

### Global webinar discussions on opportunities to align data

Findings from this review were presented as part of a global webinar attended by approximately 120 people spanning government, donors, implementing partners (both international and national), and others from all major areas of the globe. While acknowledging the importance of contextualizing measures to the context in which HIPs are implemented, the webinar was oriented toward discussing opportunities for aligning CHW and PDS data elements and definitions within and across countries to more easily track progress, foster knowledge exchange and ultimately lead to improved outcomes. Several considerations emerging from break-out discussions held as part of the webinar are summarized below.

Break-out group discussions related to CHWs started to coalesce in terms of the importance attached to the three different categories of data elements (family planning counseling, contraceptive method provision, and referrals). Specifically, there was an emerging consensus on capturing information on method provision in registers and summary forms, with a focus on the number of clients provided with a method. However, participants acknowledged the challenges of harmonizing measurement of method provision across countries given variations in CHW models (e.g., methods provided, supervisory structure) and data flows. Discussions also included support for disaggregation of method provision by client age, and by method. Some participants noted interest in additional disaggregation such as by new or returning user or other client characteristics (e.g., education, parity, ethnicity). Referrals were recommended as a process indicator for quality improvement within the facility, but seen as less of a priority for reporting into higher levels of the HMIS. There was agreement that tracking referrals (notably, referral completion while avoiding double counting) was complicated – as has been noted elsewhere ^21^ - and may mostly be useful for programmatic linkages between CHWs and health facilities. There was virtually no discussion around counseling, possibly signaling it also being largely viewed as a process indicator.

For PDS, the discussions steered away from specific data elements towards considerations for enabling private sector reporting. Participants shared that many pharmacists and drug shop operators do not see a value to reporting into the HMIS. Several participants noted that the added time burden and potential concerns around undue disclosure of client information and confidentiality of their operations could also affect willingness of pharmacies and drug shops to report. A few participants shared examples of structural mechanisms that build incentives for accountability and offer opportunities to enforce reporting. One is related to the supply chain, with private pharmacies in Zimbabwe being motivated to report because they obtain contraceptive commodities at a discounted rate from the national procurement agency. Another is regulatory, with the Pharmacy Council of Nigeria considering making reporting by PDS a requirement for annual licensing. While several of these points echo what has been reported elsewhere, ^1^^,^^7^^,^^8^ the discussions underscore tremendous appetite for continued exchanges on experiences and lessons learned.

Lastly, participants recognized the complexities of health data ecosystems and the need for context-specific solutions, which could span from integrating community-based data into the facility-based reporting chain to a dedicated community health management information system. Uganda is pursuing submission of data by drug shop operators to nearby health facilities for direct integration into the facility-based HMIS. After seeing the results of the review of tools in Uganda during a national dissemination meeting, the Commissioner for Pharmaceuticals recently committed to considering revisions of facility summary forms in the next HMIS review cycle to allow recording data from drug shops separately from those of other community-based actors and health facilities. Another example shared during the webinar came from Zimbabwe. Different systems were in place to collect family planning routine data, including data for pharmacies, and the government chose to integrate them rather than have different entities reporting into DHIS2. This was achieved through the Health Information Exchange, an interoperable and integrated platform built outside of DHIS2 that pulls family planning data from the different systems to create a central repository. ^22^

### Strengths and limitations

The context of this review brings some strengths and limitations. While the review of data collection and reporting tools was limited to five countries, the webinar discussions increased our confidence to suggest relevant considerations for advancing measurement of CHW and PDS family planning activities. The focus of this review was on how to better enable reporting of CHW and PDS family planning data within the national HMIS, but we recognize that discussions on potential revisions to data elements and data flows take place in a larger context. The realities of implementation of community-based programs are broader than family planning alone, and DHIS2 is a data management system for other health domains and, increasingly, other sectors. ^23^

Since the review was completed, the global financing landscape for health information systems has changed substantially following the termination of most USAID contracts and reductions in official development assistance by several donor countries. These changes are likely to affect countries’ capacity to maintain, update, and strengthen HMIS, including implementation of new reporting tools, indicator revisions, digital platforms, and data quality activities. The findings and recommendations presented in this paper remain relevant for informing measurement approaches, but the pace and feasibility of implementation will depend on country resources and the evolving support available for national information systems.

## Conclusion

Access to routine information on CHW and PDS family planning activities through national HMIS can allow program managers to have a comprehensive view of family planning programming in their country and inform more targeted decisions on implementation and scale-up of these two HIPs and of community-based programs. Our five-country review of CHW and PDS registers and summary forms and associated data flows and the subsequent discussions as part of a global webinar provide insight into current data availability and point at possible next steps to continue advancing measurement.

For CHWs, there is interest and opportunities to align data elements and the definitions of indicators across settings. A tentative recommendation emerged for an indicator focused on method provision, and for suggesting additional indicators on counseling and referrals but leaving their use at the discretion of countries. Variations in data flows across countries may require formulating different versions of these indicators. For settings where reporting of community-based and facility-based data is combined, future efforts should also consider advocating for CHW- and PDS-specific indicators that are agreed upon nationally.

For PDS, data availably is currently very limited. This calls for reinforcing the value of data, and of reporting into a central, accessible system. There is also a clear need to continue documenting ways that countries are attempting to overcome challenges in enabling reporting and to foster peer-to-peer learning on successful approaches for incentivizing reporting and integrating private sector data, inclusive of family planning, into national HMIS.

These conversations can and should inform the evolution of HMIS in low- and middle-income countries, which are likely to become more sophisticated over time, enabling more complex data capture and analysis that will support improved decision-making around family planning programs.

## Ethical oversight and research ethics

Based on the purpose, design, and conduct of the webinar, the webinar and any related discussions do not meet the regulatory definition of research and therefore were not subject to human subjects research oversight or research data-sharing requirements.

Under the U.S. Department of Health and Human Services regulations (45 CFR 46), research involves a *systematic investigation designed to develop or contribute to generalizable knowledge.* The webinar activities described do not meet this definition and are more accurately characterized as non-research dissemination, stakeholder engagement, and programmatic dialogue conducted outside the scope of the project.

Accordingly, the webinar did not generate research data, and participant contributions during the webinar were not collected, stored, or managed as research information. As such, there are no regulated research data associated with this activity to be shared.

## Data Availability

Summaries of analyzed tools available on Figshare: 10.6084/m9.figshare.32135878. ^24^ Data are available under the terms of the Creative Commons Attribution 4.0 International license (CC-BY 4.0).
